# Revolutionary technology of low-carbon chemical processes

**DOI:** 10.1093/nsr/nwad132

**Published:** 2023-05-13

**Authors:** Suojiang Zhang, Xiangping Zhang, Chunyan Shi, Ke Wang, Lei Yuan

**Affiliations:** Institute of Process Engineering, Chinese Academy of Sciences, China; Longzihu New Energy Laboratory, Zhengzhou Institute of Emerging Industrial Technology, China; College of Energy Science and Technology, Henan University, China; Institute of Process Engineering, Chinese Academy of Sciences, China; Institute of Process Engineering, Chinese Academy of Sciences, China; Institute of Process Engineering, Chinese Academy of Sciences, China; Institute of Process Engineering, Chinese Academy of Sciences, China

## Abstract

This perspective depicts a green hydrogen and green electricity-driven low-carbon future for chemical industry, which requires revolutionary technologies from feedstock replacements, catalyst and reactor innovations to integrated intelligent systems.

The chemical industry is one of the key sectors in China. Most chemical processes heavily rely on fossil fuels as energy resources and feedstocks, which inevitably emits a huge amount of CO_2_. With the global initiative and actions of carbon neutrality, the energy revolution of renewable energy replacing fossil fuels is further promoted towards the ultimate goal that fossil fuels will be fully replaced in the future [1]. The ongoing fundamental changes of energy structure and raw materials along with this energy revolution are most significant for chemical processes, which urgently require the development of innovative low-carbon technologies for chemical production. To fulfill this ultimate goal, a revolutionary theory and knowledge framework of chemical processes needs to be rebuilt to match the fundamental change of the driving force behind these processes: from thermo-driven to green electricity–driven. Green electricity and green hydrogen are clean and non-carbon energy carriers, which can be easily coupled with various processes. Driven by green electricity and green hydrogen, combining CO_2_, H_2_O, biomass and other renewable materials, a comprehensive revolution at multiple scales from molecular unit to system will reconstruct a low-carbon chemical industry (Fig. 1).

**Figure 1.**
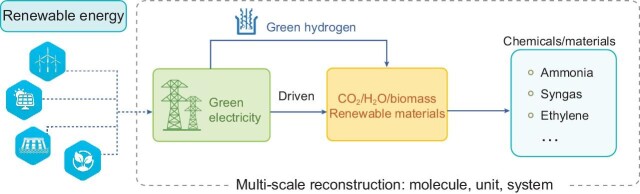
Green hydrogen– and green electricity–driven chemical process system.

Chemical processes are very diverse and complex. Ammonia, syngas and ethylene are the cornerstones of the modern chemical industry with bulk production quantity and huge amount of CO_2_ emission. Herein, we take these three typical products as examples to shed light on how chemical processes are re-engineered by green hydrogen and green electricity coupling with renewable feedstock materials.

## GREEN HYDROGEN/ELECTRICITY–DRIVEN AMMONIA SYNTHESIS

Ammonia is an important basic chemical. For now, most ammonia is used to produce fertilizers. In the context of carbon neutrality, it is also deemed as a promising way to realize hydrogen storage and transportation. Traditionally, the hydrogen and nitrogen feedstock productions for ammonia synthesis are energy and carbon-emission intensive, and the ammonia synthesis process requires a harsh condition (400–450°C, 10–15 MPa) [2]. Ammonia synthesis using green hydrogen and green electricity as feedstock and energy source, respectively, can theoretically eliminate the corresponding carbon emission. In the near future, the Haber-Bosch (H-B) process directly coupled with green hydrogen is expected to be the prevalent green ammonia synthesis technical route at the industrial scale [3]. To reduce the reaction temperature and pressure of the H-B process, and also facilitate coupling with water electrolysis hydrogen production, the most critical challenge is to achieve an efficient low-temperature N_2_ activation applicable at a large scale. For the long-term future, direct conversion of N_2_ and H_2_O to ammonia is believed to be the greenest process. However, to date, the electrochemical N_2_ reduction with H_2_O at a wide range of temperatures all exhibited production rates at nmol cm^−2^ s^−1^, which was far from the requirement of practical production [4]. Recently, lithium has been used to mediate the room-temperature N_2_ activation and NH_3_ production at a promising rate and efficiency [5]; while this process still consumed alcohols rather than water as proton sources. More investigations are needed to develop new catalysts as well as novel mechanisms to achieve ammonia synthesis in mild conditions.

## GREEN ELECTRICITY–DRIVEN SYNGAS PRODUCTION

Syngas (CO/H_2_), known as the foundation of the synthesis industry, is mainly obtained through coal gasification or natural gas steam reforming. The electroreduction of CO_2_ and H_2_O to syngas driven by green electricity fundamentally reshapes traditional thermocatalytic syngas production, which will be beneficial in order to realize carbon neutrality [6]. The key of CO_2_ electroreduction to syngas lies in the design of high-efficiency electrocatalytic systems and the scale-up of CO_2_ electrolyzer. In order to accelerate the industrial applications of CO_2_ electroreduction technology, many companies such as Siemens CO_2_ CERT Co., Ltd have made great efforts in the scale-up of reactors [7]. Among them, Carbon Energy Technology in China has established a pilot plant with a CO_2_ processing capacity of 30 t/a in Inner Mongolia Yitai Chemical Co., Ltd. However, CO_2_ electroreduction technology is still limited by the serious hydrogen evolution reaction (HER) and the long-term stability of the system. We have proposed an effective strategy to address these challenges by using novel ionic liquids instead of traditional aqueous electrolytes. For example, we prepared a Mn single atom catalyst in ionic liquid electrolyte, and achieved CO selectivity >90% in a wide overpotential range and significantly suppressed HER [8]. Furthermore, we established a large-scale CO_2_ electroreduction device with electrode active area of 495 cm^2^ using ionic liquid electrolytes in CO_2_ electroreduction technology. In a continuous operation, the CO selectivity in ionic liquid electrolyte maintained excellent stability with a high CO generation rate of 1.7 L h^−1^, showing an attractive prospect [9].

## CO_2_ REDUCTION TO PRODUCE ETHYLENE

C_2_H_4_, as one of the most produced chemicals in the world, is an important symbol of a country's petrochemical development level. The direct conversion of CO_2_ into high value-added chemical C_2_H_4_ at ambient temperature and pressure using green electricity can not only effectively mitigate the greenhouse effect, but also alleviate the energy crisis caused by the depletion of fossil energy. In addition, driven by green electricity, CO_2_ and H_2_O can be reduced to C_2_H_4_, which is the central chemical of the plastics industry. The CO_2_ electroreduction to C_2_H_4_ is a complex proton coupled electron transfer process. The regulation of highly efficient catalytic materials and reaction microenvironment can facilitate the CO_2_ to C_2_H_4_. For instance, Sargent *et al.* synthesized a novel catalytic material of Cu-based ionomer bulk heterojunction, and achieved a 60% C_2_H_4_ selectivity at a current density of 1.55 A cm^−2^, which exhibited great application prospects [10]. Although the yield and Faradaic efficiency of electrocatalytic C_2_H_4_ production have already reached a reasonable level, the biggest challenge to realize its industrialization is to further increase the single-pass conversion of CO_2_ and scaling-up the equipment.

To achieve a revolutionary low-carbon or carbon-neutral chemical industry, more and more low-carbon processes driven by green electricity and green hydrogen are also being researched—for example, the processes for aromatics, alcohols, plastics and fuels production, etc. Using green hydrogen and green electricity to power the conversion of biomass as renewable feedstocks to produce bio-chemicals is another effective strategy to reduce CO_2_ emission. The main challenges in developing these revolutionary low-carbon technologies lie in the design of new catalysts, the developments of electricity-driven reactors, the integrated intelligent systems as well as reconstructing the whole process to adapt to the renewable energy system.

## PERSPECTIVES

Chemical processes are not isolated and need systematic engineering. In the future, reliable and low-cost hydrogen storage and energy storage technologies will be developed to realize the cross-scale connection and green energy transmission between chemical industry and new energy. An intelligent system for chemical processes is also indispensable. Its role is to coordinate the energy production, storage, regulation and utilization, which supports stable, continuous and safe low-carbon chemical production. Green hydrogen and green electricity provide a critical opportunity to promote the realization of carbon neutrality in China.
